# 

**Published:** 1875-09

**Authors:** 


### Contents of September Number.

### ORIGINAL.

ART. I.—The Cornea in Health and Disease. By F. W. Abbott, M. D.... 41
ART. II.—Abstract of the Proceedings of the Buffalo Medical Association August 3d and September 7th. By E. N. Brush, M. D., Secretary..... 49
ART. III.—The Influence of Medical Studies Upon Religious Belief. By Theodore Dimon, M. D........ 54

### MISCELLANEOUS.

The Experiences of an Unsuccessful Practitioner........ 59
Urinary Retention........ 66

### MEDICAL NOTES.

Notes on Microscopy. By Geo. E. Blackham, M. D........ 66
Notes on Obstetrics and Gynæcology. By E. N. Brush, M. D........ 70

### EDITORIAL DEPARTMENT.

Homeopathy in the Michigan University........ 73
An Unmitigated Quack........ 77
Meeting of the American Pharmaceutical Association........ 78

BOOKS REVIEWED:

A Manual of Diet in Health and Disease. By Thomas King Chambers, M. D........ 79
A Report on the Hygiene of the United States Army........ 79

Books and Pamphlets Received,........ 80

